# Binge Watching during COVID-19: Associations with Stress and Body Weight

**DOI:** 10.3390/nu13103418

**Published:** 2021-09-28

**Authors:** Anahys H. Aghababian, Jennifer R. Sadler, Elena Jansen, Gita Thapaliya, Kimberly R. Smith, Susan Carnell

**Affiliations:** 1 Division of Child & Adolescent Psychiatry, Department of Psychiatry & Behavioral Sciences, Johns Hopkins University School of Medicine, Baltimore, MD 21287, USA; jsadler4@jhmi.edu (J.R.S.); elena.jansen@jhmi.edu (E.J.); gthapal2@jhmi.edu (G.T.); susan.carnell@jhmi.edu (S.C.); 2 Department of Psychiatry & Behavioral Sciences, Johns Hopkins University School of Medicine, Baltimore, MD 21287, USA; kimberly.smith@jhmi.edu

**Keywords:** binge watching, COVID-19, stress, BMI, eating behavior

## Abstract

Binge watching is becoming increasingly common and may impact energy balance and body weight. The COVID-19 pandemic has created conditions conducive to binge watching and increased stress. We investigated relationships between COVID-related stress and binge watching behaviors, and potential variation in this relationship by body weight. Adults (*n* = 466) completed a cross-sectional online survey assessing binge watching behaviors during and before the pandemic, COVID-related stress, and body weight. Participants reported an increase in binge watching frequency from before to during the pandemic (F_1,401_ = 99.970, *p* < 0.001), with rates of high binge watching (“3–4 times per week” to “3 or more times per day”) increasing from 14.6% to 33.0%. Binge watching episode duration increased from 3.26 ± 1.89 h to 3.92 ± 2.08 h (*p* < 0.001). The increase in binge watching frequency was greatest in individuals with obesity and high stress (F _4,401_ = 4.098, *p* = 0.003). Participants reporting high stress reported higher frequency of eating while binge watching, as well as higher levels of negative emotional triggers, consequences to binge watching, and lack of control over binge watching (all *p* < 0.001). Our results show that binge watching increased during the pandemic, with greater increases among individuals reporting higher COVID-related stress, especially those with obesity, and concomitant effects on eating, and highlight a need for interventions to minimize the obesogenic impact of binge watching during the pandemic.

## 1. Introduction

The COVID-19 pandemic and associated lockdown measures have disrupted daily life and impacted health-related behaviors. Sedentary behaviors, such as TV viewing, have increased during the pandemic [1,2], especially as lockdown measures force people to stay at home. Stress has also increased across the population compared to before the pandemic [3]. Stress is associated with increased TV viewing, suggesting that individuals were watching TV to cope with stress [4]. In particular, binge watching, defined as watching multiple episodes of TV programming in rapid succession, may be used to escape negative emotions. However, binge watching is also related to negative effects on psychological well-being [5,6,7] and is associated with symptoms of other behavioral addictions, including problematic TV internet/computer use, gambling, and social media addiction [5]. Excessive binge watching has been correlated with neglect of duties, social problems, and negative health-related consequences including impaired sleep [6,7]. Further, binge watching can contribute to obesity, since it is a sedentary behavior and TV watching is often accompanied by eating [8,9]. Since stress and obesity often co-occur [10], and obesity is independently associated with TV viewing [11,12], it is possible that individuals with obesity are more vulnerable to stress-induced binge watching.

Several studies have reported increases in TV watching [1,2,13] and stress [14,15,16,17,18] during the pandemic. In a sample of 1639 Italian adults, 70.8% reported an increase in TV viewing during the pandemic [13]. A survey of 38,353 Brazilian adults showed increases in the number of people who did not meet national recommendations for physical activity and engaged in high TV and computer/tablet usage (4+ h per day) during the pandemic as compared to before the pandemic [2]. Further, the increase in sedentary behavior was associated with higher odds for negative mental health outcomes, including loneliness, sadness and anxiety, and depression [1]. However, no study has directly examined the association between stress and binge watching or explored the role of body weight in this relationship. We therefore aimed to investigate the effect of COVID-related stress on changes in binge watching behaviors during the pandemic in US adults. In addition to questions about binge watching frequency and duration, we adapted a validated binge eating instrument (Questionnaire on Eating and Weight Patterns-5, QEWP-5, [19]) to capture binge watching behaviors representing the pathological and compulsive aspects of binge watching. Because past research has demonstrated sex differences in stress-related eating [20,21] and sedentary behaviors [22], we additionally tested the effect of sex differences in binge watching. We hypothesized that binge watching would increase during the pandemic, and that higher COVID-related stress and elevated body weight would be associated with greater increases in binge watching frequency and duration. We further hypothesized that higher stress and body weight would be associated with greater negative binge watching behaviors, such as eating while binge watching.

## 2. Materials and Methods

### 2.1. Study Sample and Procedure

An online survey administered via Qualtrics was used to assess how the COVID-19 pandemic affected health behaviors. The survey included 484 questions and took approximately 60 min to complete. Adults ages 18 years or older were recruited to complete the survey via Amazon’s Mechanical Turk (MTurk) and social media. Adults living in New Jersey, Delaware, District of Columbia, Illinois, California, Maine, Michigan, Nebraska, New Mexico, New York, Oregon, Pennsylvania, Tennessee, and Washington were targeted to capture people living under regional lockdowns at the time of survey distribution in May 2020. MTurk users with poor survey completion metrics (<1000 completed surveys or user-approval rating below 85%) were excluded. Participants recruited via MTurk were compensated USD 6, and participants recruited on social media were entered into a gift card lottery to win one of three USD 20 Amazon gift cards. A consent statement was provided at the beginning of the survey: “Your completion of this survey will serve as your consent to be in this research study.” All measures and procedures were approved by the Johns Hopkins University Institutional Review Board.

### 2.2. Measures

#### 2.2.1. Demographics

Participants reported demographic characteristics including age, sex, education, marital status, ethnicity, race, and employment status.

#### 2.2.2. Binge Watching

Table 1 lists the binge watching questions in full. Participants self-defined binge watching in terms of the number of hours and episodes they thought they would have to watch for it to be considered a binge. Referencing their own definitions, participants reported their binge watching frequency (“(During the past month/Before the COVID crisis), how often did you binge watch TV?”) and duration (“(During the past month/Before the COVID crisis), when you binge watched, about how long did you spend binge watching?”). If participants reported “never” binge watching before and/or during the pandemic, questions on binge watching duration before and/or during the pandemic were not displayed. Binge watching groups were created based on reported frequencies of binge watching (“Never” = No Binge Watching; “1 time per week” or “2 times per week” = Low Binge Watching; “3–4 times per week” to “3 or more times per day” = High Binge Watching). Cut-off points were based on prior findings that binge watching once or twice per week was common [5].

To evaluate the frequency of negative binge watching behaviors within the past week, including the frequency of eating while binge watching, we adapted binge eating questions from the Questionnaire on Eating and Weight Patterns (QEWP-5) [19] by replacing language regarding binge eating with language regarding binge watching. For example, the QEWP-5 question “Did you usually have any of the following experiences during these episodes (eating an unusually large amount of food in a short period of time)—feeling disgusted with yourself, depressed, or feeling very guilty afterward” (with answer choices Yes and No) was changed to “During the past week, how often did you—Start binge watching because you felt depressed or sad about something?” (with answer choices Never, Rarely, Sometimes, Often, and Always). Questions were completed with reference to *during*, and not *before*, the pandemic. We chose to adapt items from the QEWP-5, since its goal is to capture potentially pathological behaviors, and we wanted to assess pathological bingeing behaviors that were comparable to those seen around eating in order to facilitate future investigations of commonalities between binge watching and binge eating. We changed the response options from yes/no to a 1–5 Likert scale (never to always) to capture a wider variety of behavioral responses. Our adapted items allowed us to examine how individuals differed in binge watching behaviors by BMI group and COVID-related stress level.

#### 2.2.3. COVID-Related Stress

Participants responded to the question “How stressed are you about the following in relation to the COVID crisis” for 16 pandemic-related factors including finances and availability of resources, health and healthcare concerns, job and career concerns, and interpersonal concerns within and outside the home. The ratings (range = 1–5) were averaged over the 16 items into a mean score. Mean stress scores have been linked to child [23] and adult [24] intake, as well as altered feeding behaviors in parents and heightened appetite in adults [23,25]. Stress tertiles were created by splitting the ordered distribution into thirds, resulting in groups reporting low (range = 1–2.21), moderate (range = 2.25–3.09), and high (range = 3.13–5) stress levels.

#### 2.2.4. Body Weight

Participants provided self-reported height and weight, from which BMI scores (kg/m^2^) were calculated. Standard BMI cut-offs [26] were used to form BMI groups (healthy weight: (18.00–24.99 kg/m^2^), overweight (25.00–29.99 kg/m^2^), and obesity (BMI ≥ 30.00 kg/m^2^)).

### 2.3. Data Analysis

All statistical analyses were conducted using SPSS 26 (IBM Corp., Armonk, NY, USA) or RStudio (R version 4.0.3, R Studio Team, Boston, MA, USA).

Descriptive statistics were run on the binge watching variables (frequency, duration, binge watching group) and the 10 potentially negative binge watching behaviors (Table 1), as well as on demographic variables, which were compared across stress tertiles. Responses ± 3 SD of the mean for binge watching duration and frequency and BMI variables were considered extreme outliers and coded as missing data. The binge watching duration variable was normally distributed, allowing use of parametric tests. The binge watching frequency and 10 negative binge watching behavior variables were non-normally distributed, so nonparametric tests were used. A paired samples t-test was conducted to compare binge watching duration between the two time points. Sex differences among stress tertiles, BMI group, and binge watching group were examined via Chi-square tests, and sex differences in stress scores and binge watching duration were examined using independent sample *t*-tests.

To examine associations between binge watching during the pandemic and COVID-related stress, we ran bivariate analyses between the two main binge watching variables (frequency and duration) and mean COVID-related stress scores. We then stratified our sample by sex and reran these tests to examine sex differences in the association between binge watching and stress.

To examine stress tertile and BMI group differences in the change in binge watching duration from before to during the pandemic, a repeated measures Analysis of Variance (ANOVA) with time as the within-subject factor, and stress tertile and BMI group as the between-subject factors, was used. The r package ‘ARTool’ (version 0.10.8) [27] was used to perform the aligned rank transform for nonparametric factorial ANOVA to examine main effects and interactions of stress tertile (between-subject), BMI group (between-subject), and time (within-subject) on binge watching frequency.

Kruskal–Wallis tests were used to examine stress tertile differences in the 10 negative binge watching behaviors, with Mann–Whitney post hoc testing to probe differences between tertiles. To explore potential differences by BMI group in the effect of stress on the frequency of eating while binge watching, we stratified our sample by BMI group and reran these tests.

Between-group comparisons of descriptive characteristics and analyses of binge watching frequency and duration were completed using a significance threshold of uncorrected *p* < 0.05. Tests of between-group differences in binge watching behaviors were Bonferroni-adjusted for multiple comparisons with a significance threshold of *p* < 0.005.

## 3. Results

### 3.1. Sample Characteristics

The survey was initiated by 579 adults. Out of this total, *n* = 113 omitted at least one of the binge watching questions, and were therefore excluded from analyses, resulting in a final sample of *n* = 466.

Table 2 shows sample characteristics. Chi-square analysis revealed that COVID-related stress tertiles significantly differed by sex (χ^2^(2, *n* = 466) = 12.061, *p* = 0.002), with females having a higher mean COVID-stress score (M = 3.02, SD = 0.92) compared to males (M = 2.64, SD = 0.99) (t(464) = −3.015, *p* = 0.003). Additionally, Chi-square analysis revealed that BMI group distribution significantly differed by sex, such that more males were in the overweight group, while more females were in the healthy weight group (χ^2^(2, *n* = 412) = 14.92, *p* = 0.001). However, BMI did not differ between the three stress tertiles (F_2,418_ = 0.598, *p* = 0.550).

### 3.2. Binge Watching

#### 3.2.1. Self-Definition

Our participants defined binge watching duration as watching TV for a mean of at least 3.82 h (SD 1.64). *N* = 44 (9.4%) defined binge watching as watching < 2 h, *n* = 340 (73.0%) as 2–4 h, *n* = 58 (12.4%) as 4.5–6.5 h, and *n* = 24 (5.1%) as 7+ h. Binge watching, in terms of number of episodes, was defined as watching a mean of 4.55 episodes (SD 2.53). *N* = 10 (2.1%) defined binge watching as watching less than 2 episodes, *n* = 353 (75.8%) 2–5 episodes, and *n* = 102 (21.9%) 6+ episodes.

#### 3.2.2. Frequency—Before and during the Pandemic

The distribution of binge watching frequency before vs. during the COVID-19 pandemic is displayed in Table 3. Out of the 194 subjects who did not binge watch before the pandemic, 38.7% started during the pandemic (86.7% of those became low binge watchers, 13.3% became high binge watchers). Of those who were low binge watchers before the pandemic, 7.8% stopped during the pandemic, 47.1% remained low binge watchers, and 45.1% became high binge watchers. Of those who were previously high binge watchers, 76.5% remained high binge watchers and 23.5% became low binge watchers. A repeated measures ANOVA found that binge watching frequency significantly increased during the pandemic (F = 99.970, *p* < 0.001).

Binge watching frequency did not differ by sex before ($\chi 2\left(7,\;n=466\right)=7.331,\;p=0.395$) or during ($\chi 2\left(7,\;n=466\right)=4.020,\;p=0.777$) the pandemic. The proportion of participants in each binge watching group also did not differ by sex before ($\chi 2\left(2,\;n=466\right)=0.546,\;p=0.761$) or during ($\chi 2\left(2,\;n=466\right)=1.969,\;p=0.374$) the pandemic.

#### 3.2.3. Duration—Before and during the Pandemic

**Figure 1:**
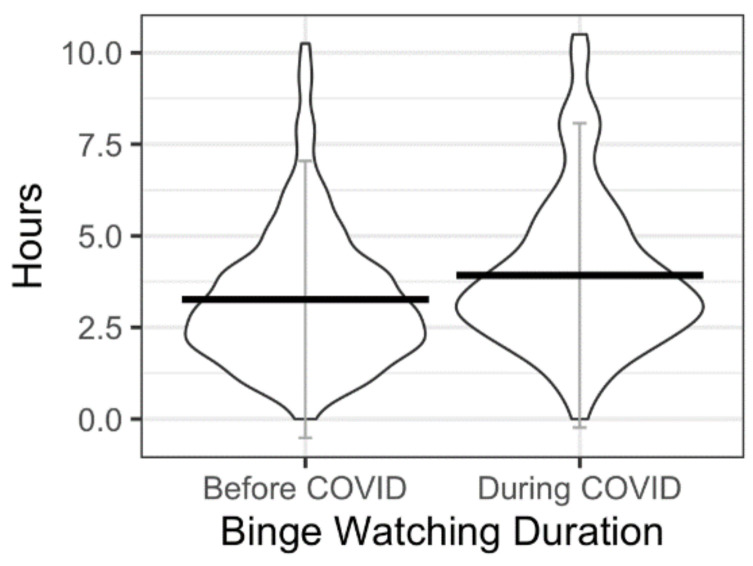
Binge watching duration before and during the pandemic.

Out of the sample, *n* = 264 (*n* = 161 females) reported their approximate binge watching duration during the pandemic and *n* = 221 (*n* = 132 females) reported their approximate binge watching duration before the pandemic. Within the reduced sample reporting data both during and before the pandemic, there was a significant difference in duration before (M = 3.26 h, SD = 1.89 h) compared with during the pandemic (M = 3.92 h, SD = 2.08 h); (t(195) = −4.883, *p* < 0.001; Figure 1). Duration was higher in females at both time points, but the mean differences in binge watching duration between the sexes were not significant before (t(219) = −0.519, *p* = 0.615) or during the pandemic (t(262) = −1.281, *p* = 0.201).

### 3.3. Relationships of Stress and Body Weight with Binge Watching Frequency and Duration before and during the Pandemic

#### 3.3.1. Frequency

Bivariate analyses revealed a significant positive correlation between mean COVID-related stress and binge watching frequency (rho(466) = 0.315, *p* < 0.001), such that greater frequency during the pandemic was associated with higher stress. When restricting analyses within sex, the association remained significant for both males (rho(169) = 0.397, *p* < 0.001) and females (rho(297) = 0.255, *p* < 0.001).

**Figure 2:**
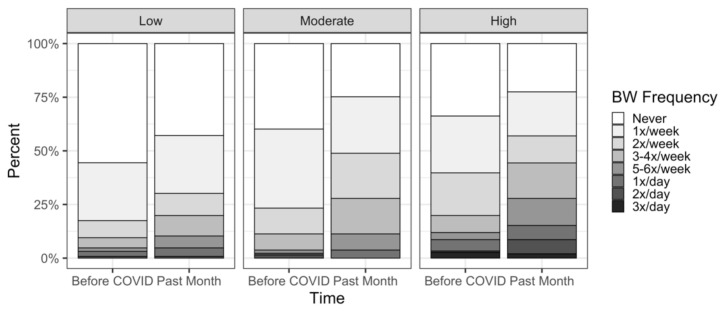
Binge Watching Frequency by Stress Tertiles.

Multivariate analyses revealed a significant main effect of stress tertile on binge watching frequency (F_2,426_ = 16.45, *p* < 0.001), such that those in the high stress tertile had higher frequency compared with the low (*p* < 0.001) and moderate (*p* = 0.05) stress tertiles, and those in the moderate stress tertile had higher frequency compared to the low stress tertile (*p* = 0.01). We also saw a stress x time interaction (F_2,401_ = 7.89, *p* < 0.001), indicating that the change in frequency differed among stress tertiles (Figure 2).

**Figure 3:**
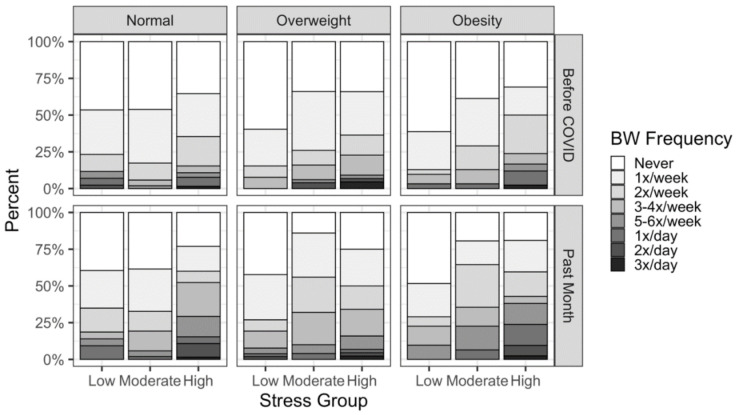
3-way Interaction between Binge Watching Frequency, BMI Groups, and Stress Tertiles.

There was no main effect of BMI group (F_2,401_ = 1.31, *p* = 0.271), but a significant three-way interaction between time, stress tertile, and BMI group emerged (F_4,401_ = 4.098, *p* = 0.003) (Figure 3).

#### 3.3.2. Duration

Duration of binge watching during the pandemic was positively correlated with COVID-related stress (r(267) = 0.164, *p* = 0.007). This positive association became non-significant when the analysis was restricted to only male respondents (r(104) = 0.161, *p* = 0.102) but remained significant when examining females only (r(163) = 0.164, *p* = 0.037).

The multivariate analysis did not reveal a main effect of stress tertile (F_2_ = 2.060, *p* = 0.131) or BMI group (F_2_ = 1.617, *p* = 0.202) on binge watching duration, and the three-way interaction of stress tertile, BMI group, and time was non-significant (F_4_ = 1.538, *p* = 0.194).

### 3.4. Negative Binge Watching Behaviors during the Pandemic

A portion of the sample, *n* = 331 (*n* = 214 female), reported on their experience of negative binge watching behaviors within the past week. Out of the 10 negative binge watching behaviors, eating while binge watching and binge watching due to boredom were the most commonly practiced in our sample, with only 9.4% and 9.9% of the sample, respectively, reporting “never” engaging in the behaviors in the past seven days (Table 4).

#### 3.4.1. Eating While Binge Watching

**Figure 4:**
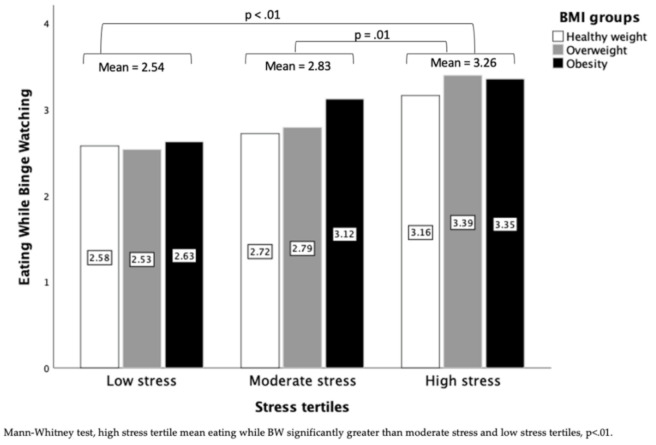
Mean frequency of eating while binge watching across stress tertiles and BMI groups.

Frequency of eating while binge watching differed by stress tertile (χ^2^(2) = 23.116, *p* < 0.001), with the high stress tertile having a higher frequency compared with the low (*p* < 0.001) and moderate (*p* = 0.001) stress tertiles (Figure 4). The number of participants reporting often/always eating while binge watching was greatest in the high stress tertile (high: 45.1%, *n* = 64; moderate: 24.8%, *n* = 23; low 9.6%, *n* = 13). After stratifying the sample by BMI group, stress effects on frequency of eating while binge watching remained in all groups: healthy weight (p-uncorrected = 0.005), overweight (p-uncorrected = 0.007), and obesity (p-uncorrected = 0.022); however, only the effect in the overweight group remained significant when controlling for multiple comparisons.

#### 3.4.2. Other Negative Binge Watching Behaviors

The frequency of other negative binge watching behaviors significantly differed by stress tertile (all *p* < 0.001; Table 5). All effects survived correction for multiple comparisons. Mann–Whitney tests revealed that the high stress tertile had higher frequencies of all behaviors compared with the low stress and moderate stress tertiles. For all items except eating while binge watching and binge watching due to boredom, the moderate stress tertile also had higher frequencies compared with the low stress tertile (all p-uncorrected < 0.05).

## 4. Discussion

We investigated how binge watching changed during the COVID-19 pandemic and how stress and BMI related to binge watching behaviors. In a large sample of US adults, we found that the frequency and duration of binge watching increased during the COVID-19 pandemic as compared to before the pandemic. We also found that increases in frequency and duration were greater among respondents who reported higher stress related to the COVID-19 pandemic and among those with obesity.

In our sample of 466 US adults, the mean duration and frequency of binge watching increased during the pandemic. This is in line with previous findings of increased TV viewing during the pandemic [1,2,13]. However, there was substantial individual variation in change, and some individuals decreased binge watching during the pandemic. Of those who were low binge watchers before, 7.8% stopped binge watching during the pandemic, and of the high binge watchers before the pandemic, 23.5% became low binge watchers. COVID-related stress may explain some of the individual variations in binge watching behaviors. We found that higher stress was associated with a greater increase in binge watching frequency. This increase was greatest among high stress individuals with obesity and, contrary to our predictions, among healthy weight individuals. We speculate that the large increase in healthy weight individuals may be driven by the fact that those in the healthy weight and high stress groups were younger and had fewer children, which may have allowed more time for binge watching. We also saw that higher stress was associated with a higher frequency of potentially negative binge watching behaviors (e.g., eating while binge watching, starting binge watching due to depression/sadness). Results suggest that our sample may be using TV viewing to cope with negative emotions/states during the pandemic. In fact, 53.2% of our sample reported initiating binge watching because of depression or sadness, and those with higher stress scores were more likely to report this behavior. Similarly, people who use binge watching to distract or escape from negative thoughts and feelings were more likely to experience a loss of control (measured using Polish Questionnaire of Excessive Binge-Watching Behaviors) over the amount they binge watch [6,28]. Loss of control can lead to excessive binge watching, possibly resulting in neglect of duties, social problems, and health-related consequences, such as sleep problems and unhealthy eating habits [5,6,7].

In our exploration of sex differences in binge watching, we found that neither binge watching frequency nor duration differed between males and females at either timepoint, and the positive correlation of COVID-related stress with binge watching frequency and duration reached significance in both males and females. However, mean COVID-related stress scores were higher in females. Previous studies found that female binge watching is characterized by entertainment, loneliness, and social motivations [6], and that females were more likely to binge watch to reduce negative emotions or escape unpleasant affective states [28]. Our results suggest that binge watching among females may also be motivated by a desire to reduce stress.

Binge watching is a sedentary behavior associated with higher body weight and may promote obesity if adopted as a coping mechanism during stressful times [11,12,29]. Sedentary behaviors such as binge watching have been reinforced during the pandemic, with stay-at-home orders restricting opportunities for physical activity [30]. Furthermore, TV viewing is associated with increased food intake, especially of highly processed, high energy-density snack foods [9]. In accordance with this established phenomenon, 86.7% of our sample reported eating while binge watching. We did not attempt to assess the type or amount of food consumed while binge watching. However, people are often unaware of how much they eat while distracted [8], making binge watching a likely trigger of excessive consumption. We found that those in the high stress tertile ate while binge watching more frequently than those in the moderate or low stress tertiles, with this difference being most apparent in participants with overweight. While the effects did not survive correction for multiple comparisons, differences by stress tertile in those with healthy weight and those with obesity showed the same direction of effect, where increasing COVID-related stress was associated with more frequent eating while binge watching. Our findings are broadly in line with a previous study showing that heavy screen users had higher BMI and increased stress [4], and suggests that individuals with high pandemic-related stress and higher body weight may be at heightened risk of not only binge watching itself, but also of eating while binge watching. Although acute stress can reduce food intake [31], chronic stress may dysregulate the hypothalamic-pituitary-adrenal (HPA) axis, promoting food intake [32]. Eating during episodes of binge watching may be the result of chronic stress stimulating appetite. It is unclear why associations between stress and eating while binge watching were strongest in participants with overweight rather than participants with obesity. However, non-linear effects of BMI have also been observed for other eating behaviors [33]. Social desirability may be one contributor, as may be the presence of HPA axis perturbations in some individuals with obesity [34].

We observed significant relationships between COVID-related stress and binge watching, but stress did not completely explain individual variation in binge watching behaviors. Increased binge watching is likely attributable to government-mandated stay-at-home orders that were associated with increases in boredom [28]. Boredom was the most common binge watching motivation in our sample, with 86.1% of our sample reporting initiating binge watching due to boredom. Heavy screen usage can lead to poor sleep quality and reduced sleep duration as well as lower physical activity and poor dietary choices [4], so while binge watching in response to boredom may be less pathological than binge watching in response to stress, it may nonetheless negatively impact health.

Our study had several limitations. First, our survey was distributed after the COVID-19 pandemic already began, so reports of binge watching behaviors before the pandemic were retrospective and subject to recall bias. Moreover, our survey was cross-sectional, limiting our ability to draw causal conclusions. Another limitation was that BMI was calculated from participants’ self-reported body weight and height. People underestimate their weight and overestimate height [35], so BMI values may be inaccurate. Further, since self-reported height and weight were collected several months into the pandemic, BMI values might partly represent downstream effects of binge watching behaviors rather than independently ‘predicting’ effects of stress on binge watching. Since we aimed to capture the compulsive nature of binge watching, we assessed binge watching in relation to individuals’ self-perceptions of what constitutes as a binge. The majority of studies in the literature have agreed that watching 2 or more episodes of a TV defines binge watching [5,7,36,37], so we reran our analyses excluding those who defined binge watching as < 2 episodes (*n* = 10). Our results persisted, suggesting that individuals’ perceptions of a binge may be a valid way to operationalize binge watching for future research.

Our findings shed light on possible relationships between binge watching, stress, and obesity. Given the sedentary nature of binge watching and the potential to overeat while binge watching, it may be beneficial for some individuals to reflect on and consider reducing their use of binge watching to cope with stress or negative emotions. While interventions to reduce binge watching have not been widely studied, there are a number of studies that have identified successful interventions to reduce sedentary behaviors [35,36]. Methods such as the use of technology to deliver movement reminders and environmental changes (e.g., introduction of a standing desk) show promise for reducing sedentary behavior and could be applied to target binge watching specifically. There is a significant need to promote physical activity and health behaviors among the population during the pandemic, and our results suggest that recommendations to help avoid potential negative aspects of binge watching may be an important component of such education. Our findings add to previous research suggesting a pathological dimension to binge watching [38] by revealing a relationship with higher BMI and increased eating while binge watching. We propose further research on these phenomena as well as exploring the potential relationship between binge watching and binge eating.

## 5. Conclusions

Our findings add to a growing literature on binge watching by examining how stress, BMI, and their interaction relate to binge watching behaviors. Obesity is a leading public health concern [39], making it imperative to identify factors underlying obesogenic behaviors, such as binge watching. To our knowledge, this is the first study to assess binge watching behaviors using an adapted binge eating questionnaire used for clinical diagnoses. This instrument will facilitate future research investigating pathological aspects of binge watching, as well as relationships between binge watching and binge eating. Our results highlight a potential target for interventions to minimize the obesogenic impact of the ongoing COVID-19 pandemic, especially among those with high stress and elevated body mass.

## Figures and Tables

**Table 1:** Binge watching questions.

Item	Answer Choices
Definition How many hours do you think one would need to watch TV in one sitting to consider it a binge? How many episodes of a TV show do you think one would need to watch in one sitting to consider it a binge?	Hours (0–12 h in 30 min increments) Episodes (0–20)
Frequency How often did you binge watch TV? During the past month Before the COVID crisis	Never 1 time per week 2 times per week 3–4 times per week 5–6 times per week 1 time per day 2 times per day 3 or more times per day
Duration During the past month, when you binge watched, about how long did you spend binge watching? Before the COVID crisis, when you binge watched, about how long did you spend binge watching?	Hours and minutes
Negative binge watching behaviors During the past week, how often did you: Eat while binge watching? Start binge watching because you felt depressed or sad about something? Start binge watching because you felt bored? Feel bad after binge watching? Feel that binge watching interfered in your day-to-day life (e.g., work, school, household responsibilities)? Feel like you can’t stop watching or control how much you watch? Skip onto the next episode in a series more quickly than you normally would? Watch until you felt uncomfortable (e.g., tired, dry-eyed)? Watch alone because you were embarrassed by how much you were watching? Feel disgusted with yourself, depressed, or feeling very guilty after watching?	1 = Never 2 = Rarely 3 = Sometimes 4 = Often 5 = Always

**Table 2:** Sample characteristics.

Variable		Low Stress *n* = 162 *N* (%)	Moderate Stress *n* = 151 *N* (%)	High Stress *n* = 153 *N* (%)	Full Sample *n* = 466 *N* (%)
Sex *	Male	75 (46.3)	42 (27.8)	52 (34.0)	169 (36.3)
	Female	87 (53.7)	109 (72.2)	101 (66.0)	297 (63.7)
BMI	Mean ± SD	26.94 ± 5.28	27.09 ± 5.90	27.67 ± 6.17	27.21 ± 5.76
	Range	10.63–46.34	15.66–45.61	17.47–45.48	10.63–46.34
BMI groups	Healthy weight	55 (34.0)	56 (37.1)	50 (32.7)	161 (34.5)
	Overweight	58 (35.8)	47 (31.1)	42 (27.5)	147 (31.5)
	Obesity	36 (22.2)	32 (21.2)	36 (23.5)	104 (22.3)
Education	Less than 10th grade	0 (0)	0 (0)	1 (0.7)	1 (0.2)
	High school degree (GED)	12 (7.4)	9 (6.0)	8 (5.2)	29 (6.2)
	Trade school/apprenticeship	2 (1.2)	1 (0.7)	2 (1.3)	5 (1.1)
	Partial college	21 (13.0)	26 (17.2)	24 (15.7)	71 (15.2)
	2-year college	24 (14.8)	22 (14.6)	14 (9.2)	60 (12.9)
	4-year college	60 (37.0)	60 (39.7)	65 (42.5)	185 (39.7)
	Graduate degree	43 (26.5)	33 (21.9)	39 (25.5)	115 (24.7)
Marital status	Single	25 (15.4)	26 (17.2)	21 (13.7)	72 (15.5)
	Partnered/married	125 (77.2)	113 (74.8)	121 (79.1)	359 (77.0)
	Divorced/separated	11 (6.8)	10 (6.6)	10 (6.5)	31 (6.7)
	Widowed	1 (0.6)	0 (0)	1 (0.7)	2 (0.4)
	Other	0 (0)	2 (1.3)	0 (0)	2 (0.4)
Ethnicity	Hispanic or Latino	15 (9.3)	13 (8.6)	19 (12.4)	47 (10.1)
	Not Hispanic or Latino	146 (90.1)	135 (89.4)	130 (85.0)	411 (88.2)
	Don’t know	0 (0)	2 (1.3)	1 (0.7)	3 (0.6)
	Prefer not to answer	1 (0.6)	1 (0.7)	3 (2.0)	5 (1.1)
Employment status	Student	1 (0.6)	8 (5.3)	8 (5.2)	17 (3.6)
	Self-employed	9 (5.6)	9 (6.0)	6 (3.9)	24 (5.2)
	Employed part-time	5 (3.1)	25 (16.6)	15 (9.8)	45 (9.7)
	Employed full-time	124 (76.5)	78 (51.7)	98 (64.1)	300 (64.4)
	Unable to work due to disability	0 (0)	2 (1.3)	0 (0)	2 (0.4)
	Homemaker/full-time parent	19 (11.7)	19 (12.6)	16 (10.5)	54 (11.6)
	Unemployed/seeking work	2 (1.2)	10 (6.6)	9 (5.9)	21 (4.5)
	Retired	2 (1.2)	0 (0)	1 (0.7)	3 (0.6)
Essential worker	Yes	50 (30.9)	45 (29.8)	42 (27.5)	137 (29.4)
	No	112 (69.1)	106 (70.2)	111 (72.5)	329 (70.6)

* = Significant difference between groups (Chi-squares, *p* < 0.05).

**Table 3:** Binge watching frequency before and during the pandemic.

	Binge Watching Level	Before the COVID Pandemic *N* (%)	In the Past Month *N* (%)
Never	No	194 (41.6%)	135 (29.0%)
1 time per week	Low	138 (29.6%)	110 (23.6%)
2 times per week	Low	66 (14.2%)	67 (14.4%)
3–4 times per week	High	32 (6.9%)	68 (14.6%)
5–6 times per week	High	12 (2.6%)	44 (9.4%)
1 time per day	High	13 (2.8%)	24 (5.2%)
2 times per day	High	5 (1.1%)	12 (2.6%)
3 or more times per day	High	6 (1.3%)	6 (1.3%)

**Table 4:** Negative binge watching behaviors.

Item	Response	Frequency	Percent
Start binge watching because you felt depressed or sad about something?	Never	155	46.8
	Rarely	60	18.1
	Sometimes	79	23.9
	Often	28	8.5
	Always	9	2.7
Start binge watching because you felt bored?	Never	46	13.9
	Rarely	52	15.7
	Sometimes	112	33.8
	Often	97	29.3
	Always	24	7.3
Feel bad after binge watching?	Never	144	43.5
	Rarely	66	19.9
	Sometimes	71	21.5
	Often	36	10.9
	Always	14	4.2
Feel that binge watching interfered in your day-to-day life (e.g., work, school, household responsibilities)?	Never	140	42.3
	Rarely	67	20.2
	Sometimes	63	19.0
	Often	52	15.7
	Always	9	2.7
Eat while binge watching?	Never	44	13.3
	Rarely	57	17.2
	Sometimes	127	38.4
	Often	77	23.3
	Always	26	7.9
Feel like you can’t stop watching or control how much you watch?	Never	137	41.4
	Rarely	80	24.2
	Sometimes	70	21.1
	Often	34	10.3
	Always	10	3.0
Skip onto the next episode in a series more quickly than you normally would?	Never	153	46.2
	Rarely	54	16.3
	Sometimes	70	21.1
	Often	43	13.0
	Always	11	3.3
Watch until you felt uncomfortable (e.g., tired, dry-eyed)?	Never	135	40.8
	Rarely	73	22.1
	Sometimes	73	22.1
	Often	40	12.1
	Always	10	3.0
Watch alone because you were embarrassed by how much you were watching?	Never	228	68.9
	Rarely	33	10.0
	Sometimes	41	12.4
	Often	20	6.0
	Always	9	2.7
Feel disgusted with yourself, depressed, or feeling very guilty after watching?	Never	191	57.7
	Rarely	57	17.2
	Sometimes	37	11.2
	Often	24	10.3
	Always	12	3.6

**Table 5:** Frequency of negative binge watching behaviors by stress tertiles.

Items	Low Stress *n* = 78 Mean (SD)	Moderate Stress *n* = 109 Mean (SD)	High Stress *n* = 142 Mean (SD)	H
1. Start binge watching due to depression or sadness	1.38 (0.856)	1.77 (0.949)	2.54 (1.153)	64.372 *^,a,b,c^
2. Start binge watching due to boredom	2.64 (1.206)	2.86 (1.182)	3.30 (0.996)	18.256 *^,b,c^
3. Feel bad after binge watching	1.50 (.769)	1.98 (1.045)	2.54 (1.324)	36.819 *^,a,b,c^
4. Binge watching interfering with daily life	1.56 (.934)	2.06 (1.057)	2.54 (1.303)	33.467 *^,a,b,c^
5. Eat while binge watching	2.54 (1.113)	2.83 (0.970)	3.26 (1.140)	23.116 *^,b,c^
6. Cannot control binge watching	1.53 (0.849)	1.96 (1.009)	2.49 (1.213)	38.195 *^,a,b,c^
7. Skip quickly to next episode	1.54 (0.949)	1.91 (1.076)	2.57 (1.279)	41.128 *^,b,c^
8. Binge watch until feeling uncomfortable	1.63 (0.941)	2.01 (1.076)	2.52 (1.230)	31.530 *^,b,c^
9. Binge watch alone due to embarrassment	1.15 (0.605)	1.48 (0.909)	1.99 (1.232)	37.000 *^,a,b,c^
10. Feelings of disgust/guilty after binge watching	1.28 (0.719)	1.59 (0.925)	2.32 (1.340)	46.588 *^,b,c^

Kruskal–Wallis H Test, * = *p* < 0.001, ^a^ = significant post hoc difference between low and moderate stress tertile, ^b^ = significant post hoc difference between low and high stress tertile, ^c^ = significant post hoc difference between moderate and high stress tertile via Mann–Whitney Test.

## Data Availability

Data available on request due to ongoing analysis and use.
